# Experimental Techniques to Obtain the Cross-Sectional Images of Textile Yarns

**DOI:** 10.3390/ma15144726

**Published:** 2022-07-06

**Authors:** Mohamed Abdelkader, Adnan Mazari, Sumayya Zafar

**Affiliations:** 1 Department of Advanced Materials, Institute for Nanomaterials, Advanced Technologies and Innovation (CXI), Technical University of Liberec, 46117 Liberec, Czech Republic; 2 Department of Clothing, Faculty of Textiles, Technical University of Liberec, 46117 Liberec, Czech Republic; adnan.ahmed.mazari@tul.cz; 3 Department of Computer and Information System Engineering, NED University of Engineering and Technology, Karachi 75270, Pakistan; sumayya@neduet.edu.pk

**Keywords:** yarn, cross section, slice, fiber, packing density, microtome, micro-computed tomography, epoxy, grinding, polishing, twist, diameter

## Abstract

In the fabric industry, textile yarns are the fundamental building blocks. Hence, visualizing and studying yarn structure is essential to understand the structure and behavior of the fibers. Obtaining the yarn’s cross-section images is crucial in the calculations of yarn’s porosity; furthermore, a more precise expansion for the fiber’s migration can be concluded from the cross-sectional images. In this paper, three different methods (microtome, micro-computed tomography, and epoxy grinding–polishing methods) to image and visualize the yarn’s cross-section are presented. The experimental techniques are compared in terms of result useability, time of preparation, and overall outcome of the cross-sectional image. The images can be used for fiber distribution, air gap calculation, and twist analysis as well. The fiber diameter distribution of polyester yarn was measured based on the images obtained by the three different methods; the average fiber diameter measured based on the combined data from the three different methods was found to be 10.90 ± 0.30 µm.

## 1. Introduction

A textile yarn can be defined as a group of twisted fibers [1]; the shape and properties of the fibers form the overall properties of the yarn. Hence, studying and understanding the distribution and shape of the fibers across the length of the yarn is beneficial [2,3], and this information can be extracted from yarn cross-sectional slices or images. Analyzing the images of these sections can reveal the geometrical parameters, such as packing density, effective packing density, and ellipticity shape [4,5,6], which contribute to the yarn’s properties, such as tensile strength, elongation, appearance, compactness, and the overall fabric surface [7]. Obtaining detailed and high-resolution slices can reveal the details of the yarn’s structure and allow deeper analysis, modeling, and simulation of the textile structure, which can be more realistic and representative of the actual model; several groups simulated the textile yarn using digitally generated models [8,9,10].

Researchers used two methods to obtain cross-sectional images of yarns in the literature. The first method is the microtome technique [11,12], which is used to obtain a-few-micrometer-thick sliced sections of the sample (for example, the textile yarn). Then, these slices are imaged by an optical microscope [13]. Musa’s group used the rotary microtome to obtain cross-sectional images of natural fibers produced by the ring, vortex, and compact spinning methods; the work aimed to study the effect of the spinning method on the yarn’s packing density, and the group concluded that compact yarns possessed the highest packing density, while vortex had the lowest [13]. The microtome method can provide a quite clear cross-sectional image; however, obtaining a correct sequence of slices using a microtome can be a difficult and time-consuming process and, at the same time, it is a destructive method, so the scanned sample can be no longer used for other tests or scans [14].

The second method is through micro-computed tomography (µCT), in which the textile yarn can be three-dimensionally scanned and reconstructed, and the cross-sectional images or series of slices can be obtained from the reconstructed tomography dataset [15,16]. Noman’s group used µCT to investigate the cotton-ring-spun yarns; they studied the microstructure of the yarn and the fiber arrangement effects on the strength of the fiber, and the study concluded that the higher strength of yarn correlated with a higher value of fiber-migrating behavior in amplitude and intensity across the yarn and vice versa [17].

The µCT method has several advantages when compared to the traditional microtome method. µCT is a nondestructive and noninvasive imaging method where the sample preserves its original form; in addition, it obtains realistic and accurate details of the scanned object, hence, enabling digital reconstruction of a realistic model that matches the order and accuracy of the original scanned object [18,19,20]. Moreover, it allows partial segmentation of the scanned sample, so the single fibers of the yarn can be segmented and studied individually [21,22].

In this paper, in addition to the microtome method and the µCT method, we introduce a new method to obtain images of yarn cross-sections where a customized 3D holder was used to hold multiple yarn samples, then the holder was molded by epoxy resin. In order to get clear and high-quality cross-sectional images, a grinding polishing approach was used. After each grinding–polishing step, an image was taken using an optical microscope. The three methods (microtome, µCT, and epoxy grinding–polishing) were used to obtain the cross-sectional images of a polyester yarn sample, and the paper shows the practical steps for each method and a comparison between the three different methods.

## 2. Materials and Methods

A polyester yarn sample (1.4 dtex/38 mm) was used in all the 3 methods with the following parameters: yarn count: 34 Nm, twist coefficient (α): 110, and twist level: 650 Z/m.

The different methods to obtain cross-sectional images of the yarn are summarized in the following points:

### 2.1. Microtome

**Figure 1:**
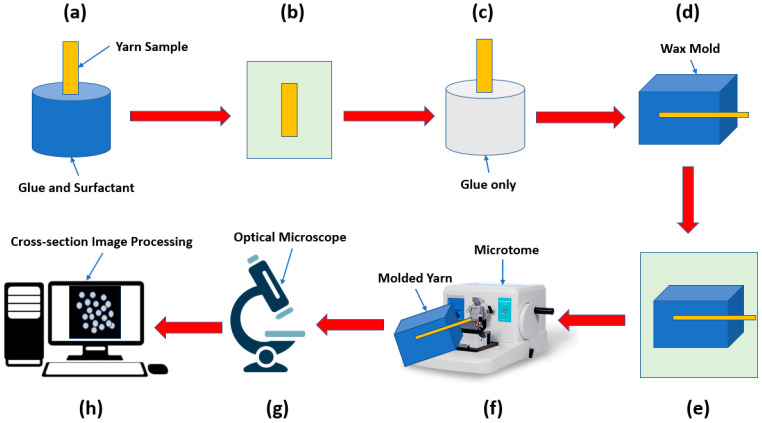
Experimental steps of microtome method: (**a**) immersion in glue and surfactant, (**b**) drying in standard atmospheric conditions for 24 h, (**c**) immersion in a glue only, (**d**) mold of bee’s wax and paraffin, (**e**) freezing at—8 °C for 24 h, (**f**) microtome slicing, (**g**) microscopic investigation, (**h**) image processing and analysis.

The first step in preparing the yarn samples was to cover the samples with the glue using different concentrations of the glue, on the first day the samples were dipped in a glue solution (glue 30% + water 70%). The same step was repeated for the second day but by immersing the samples in an 80% glue solution (glue 80% + water 20%). On the third day, the samples were covered with only glue using a small painting brush. On the fourth day, the samples were molded with wax, and then the samples were put in the freezer at a temperature of −8 °C. After that, the samples were cut into 15 µm slices using the microtome Leica RM 2155 (Leica Biosystems, Wetzlar, Germany). The slices were imaged using an optical microscope NIKON ECLIPSE E200 (Nikon Instruments Inc., Melville, NY, USA) Nikon NIS-Elements software (Laboratory Imaging s.r.o., Prague, Czech Republic) for fiber diameter analysis and measurement. Figure 1 shows the experimental steps to obtain the cross-sectional images of a textile yarn using a microtome.

### 2.2. Micro-Computed Tomography (µCT)

The textile yarn sample was scanned using Bruker Skyscan 1272 (Bruker, Billerica, MA, USA) micro-computed tomography scanning machine with the following parameters, as shown in Table 1.

**Figure 2:**
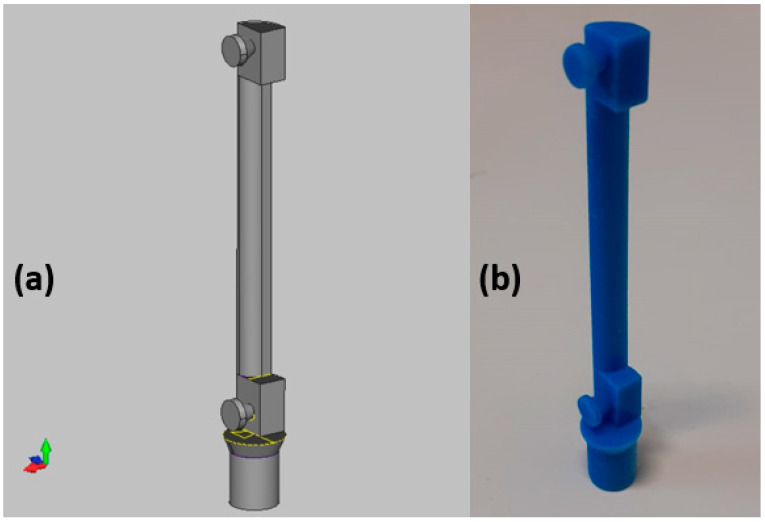
The 3D holder: (**a**) 3D design (**b**) the printed holder.

A compatible holder with the µCT scanner was designed using Autodesk Inventor Professional 2018 (Autodesk, San Rafael, CA, USA) and it was 3D printed to sustain the yarn sample in its original form and twist during the scanning process; the holder was designed to keep the sample centered geometrically so it can allow using the highest scanning resolution and at the same time reducing the scanning width as much as possible to obtain smaller datasets and faster data handling and processing. Figure 2 shows the 3D design of the holder with the compatible base that fits in the scanner holder.

The yarn sample was cut according to the following method to make sure the twist is maintained in the cut sample which assures the actual representation of the scanned structure.

In order to trap the twist inside the sample, the following steps were followed:

- Unwind a length (2–4 m) from the beginning of the yarn bobbin (that length will be disposed).
- Make a knot after that disposable length.
- Unwind the length of the sample (1–2 m) and make another knot.

**Figure 3:**
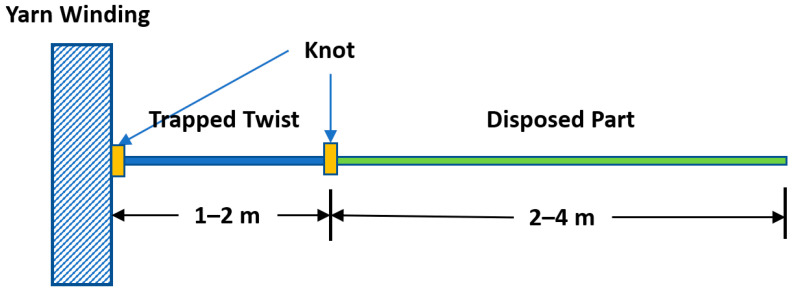
Twist trap process.

Figure 3 summarizes the twist trap process. This process should maintain the original manufacturing twist.

**Figure 4:**
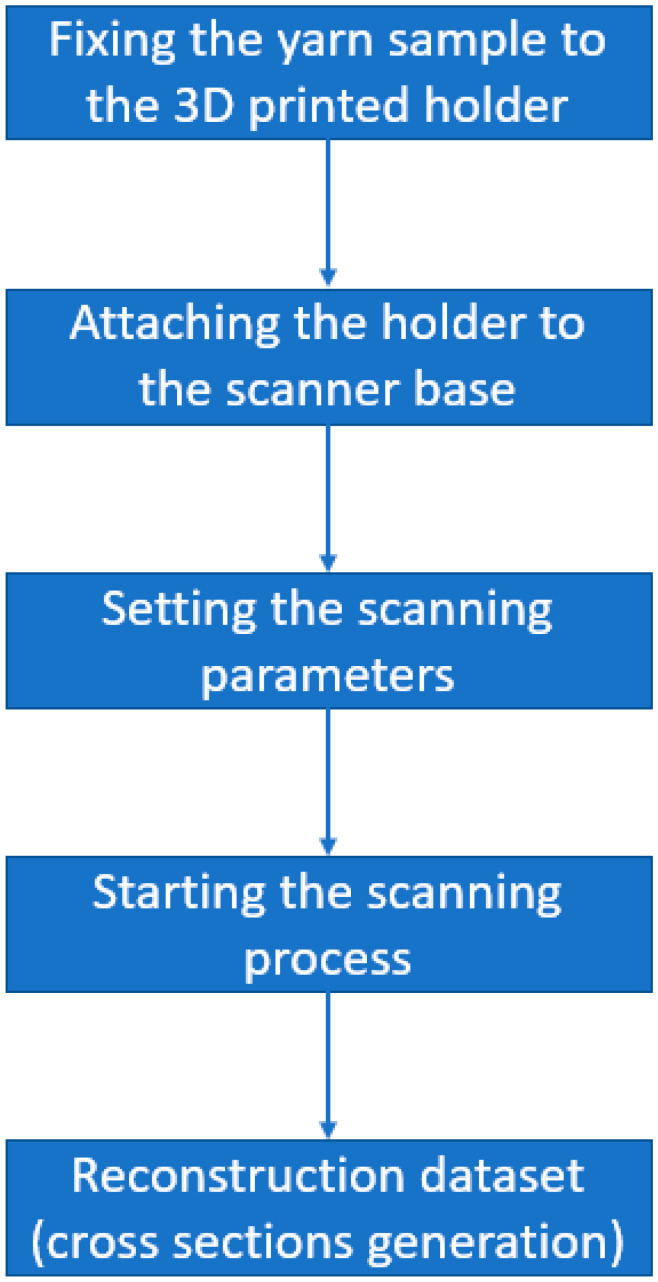
Summary of the µCT method.

Then the cut sample according to that method was attached to the 3D-printed holder, and after that the holder was put into the scanner. The previously mentioned scanning parameters were set to the scanner and the 3 mm length of the yarn was chosen as the scanning region. NRecon Bruker software (NRecon 2016, Bruker, Billerica, MA, USA) was used to reconstruct the projection images and extract the cross-sectional images (2D matrix), and when these images are stacked in a 3D matrix, it is possible to view the 3D representation of the textile yarn (a digital 3D representation of the scanned sample). Figure 4 summarizes the µCT sample scanning procedure.

### 2.3. Epoxy Grinding Polishing

**Figure 5:**
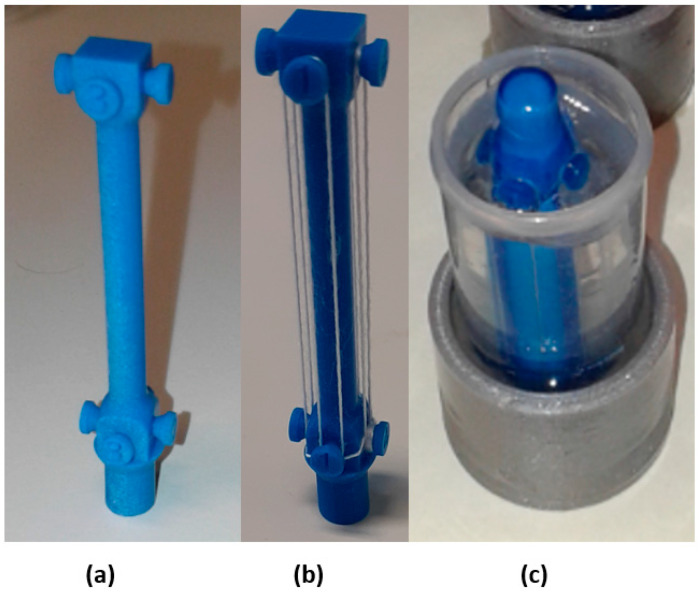
(**a**) The multiholder (3D printed), (**b**) the multiholder loaded with yarn pairs, (**c**) the prepared holder in a 3D plastic container after pouring the epoxy resin.

In this method, multiple yarns were supported using the 3D-printed multiholder, as shown in Figure 5a, to form 4 pairs of yarns (8 yarns). Yarn samples were attached to each side of the holder in pairs using the twist trap method mentioned previously; the step was repeated till covering the 4 sides of the holder. After that, the holder was attached to the plastic container using a wax gun to be fixed during the pouring and curing of the epoxy’s resin.

Epox G300 (Dawex Chemical s.r.o., Prague, Czech Republic) solution was prepared using component A (Resin) and component B (Hardener) with a ratio 4:1, respectively; the epoxy mixture was poured into the plastic container and left for 48 h to cure at room temperature (≈22 °C). A fitting cup as shown in Figure 5b (which is compatible in size with the grinding and polishing machine holder) with a diameter of 30 mm was 3D printed, and the plastic container was held inside it using an epoxy mixture. The samples were left to cure at room temperature for 48 h inside a vacuumed chamber under pressure of 0.3 Pa. The desired pressure was achieved using a 0.33 HP vacuum pump (model: VP225, VacuumChambers.eu, Białystok, Poland) to eliminate the existence of bubbles in the samples.

After the samples were cured, the sample was cut manually using a PARKSIDE manual (Parkside group, Queensland, Australia) saw to a length of 22 mm, which matches the length of the machine mold for the grinding and polishing machine. After cutting the sample to the desired length, the grinding step starts. The grinding was done in 4 steps using the Struers Tegramin-25 machine (Struers, Copenhagen, Denmark), silicon carbide grinding foils (Struers, Copenhagen, Denmark) #220, #1200, #2000 and #4000 were used for the grinding process. Silicon carbide foils number 220 and 1200 were applied to the sample for 3:30 min, while the foils 2000 and 4000 were applied for 2 min. Following the grinding process comes the polishing process; the polishing step was performed using 3 µm polishing sheet with monocrystalline diamond suspension. In other cycles of the grinding–polishing process, the grinding time was set to 10 s which corresponds to almost 15 µm thickness of the removed material, while the time for polishing was the same as the first polishing step.

**Figure 6:**
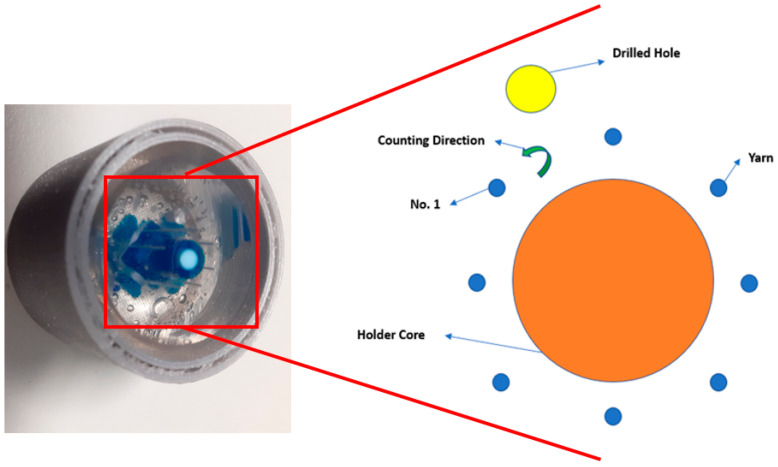
Holder marking system of multiple yarns.

After getting the samples polished, the samples were scanned with an optical microscope using backfield reflection imaging mode (images were captured at 50× optical magnification). The samples were scanned using ZIESS AXIO imager optical microscope (Carl Zeiss AG, Jena, Germany). To differentiate the different yarns under the microscope, a marking system was placed by making a hole using an electric drill. The counting of the yarns starts anticlockwise from the left yarn next to the drilled hole, as shown in Figure 6.

**Figure 7:**
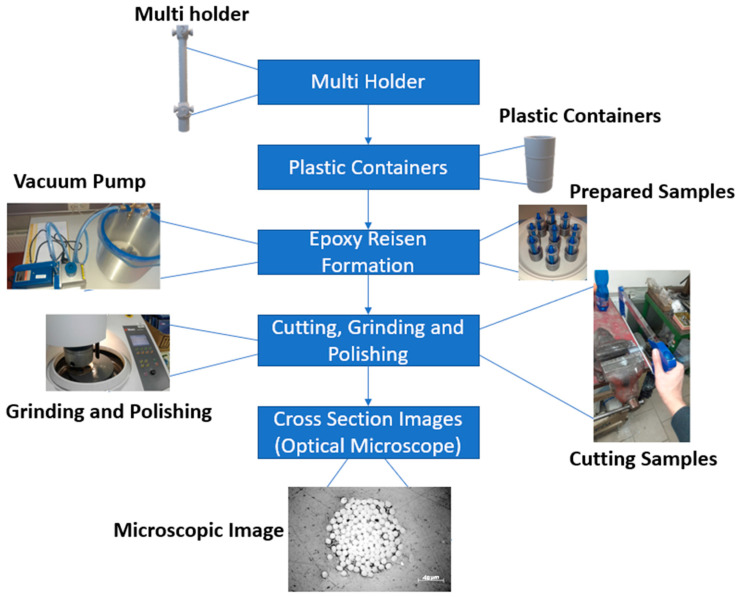
Summary of the epoxy grinding polishing method.

Figure 7 summarizes the main steps that were followed to obtain the cross-sectional images.

## 3. Results and Discussion

### 3.1. Microtome Results

**Figure 8:**
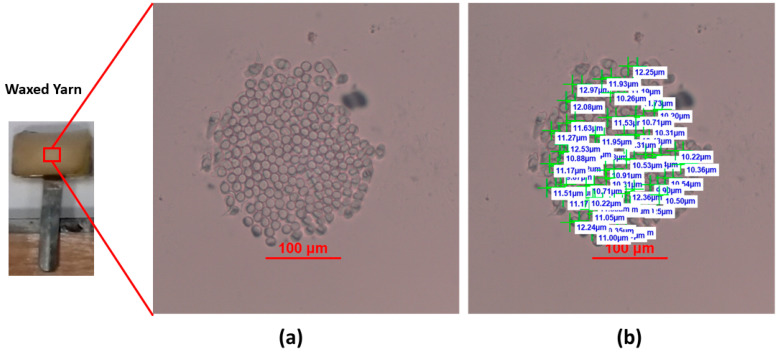
(**a**) Cross-sectional image of the polyester yarn obtained by microtome method, (**b**) individual fiber diameter measurements in the cross-section.

Figure 8a shows a cross-section of the yarn under the optical microscope, while Figure 8b shows the diameter measurements of individual fibers in the cross-section. It was found that the mean value diameter of the fibers was 10.96 ± 0.83 µm. We noticed that some of the fibers are out of focus, which means that not all of the fibers exist in the same geometrical plane (the microtome cut was not completely straight). Several studies in the literature used the microtome method to obtain the yarn’s cross-sectional images and correlate them with the yarn’s properties [23,24,25].

### 3.2. Micro-Computed Tomography (µCT) Results

**Figure 9:**
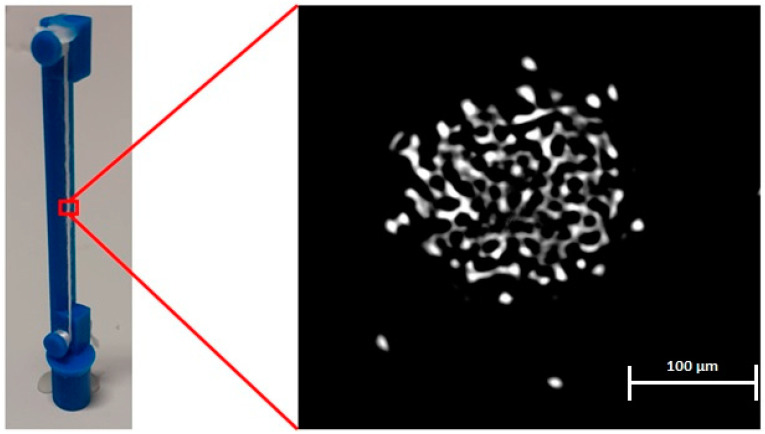
Cross-sectional image of the polyester yarn obtained by µCT.

After reconstructing the projection images using NRecon software, the dataset of cross-sections was obtained, which is a stack of sliced cross-sections. This stack of slices can construct the 3D structure of the yarn when visualized altogether in the same order as a 3D matrix. Figure 9 shows one of the obtained slices (a yarn cross-section). It was noticed that the structure of individual fibers is not that clear because of the scanning resolution, as one pixel represents one micrometer, which means that the diameter of a single fiber is represented by 10–12 pixels. A possible solution for this can be using a higher-resolution scanner that, at least, can represent the diameter of individual fibers by 22–25 pixels (scanning resolution ≈ 0.5 µm) or applying image processing algorithms on the obtained cross-sections. The fibers’ diameter distribution was measured manually by ImageJ [25], and the mean diameter obtained by µCT was found to be 10.50 ± 1.13 µm. Several studies in the literature used computed tomography to visualize and analyze different composite and textile structures [26,27].

**Figure 10:**
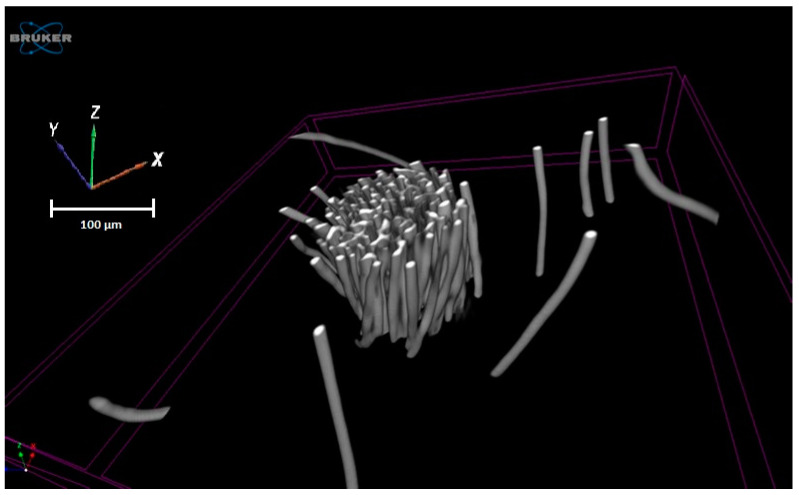
3D reconstruction of the polyester yarn obtained by µCT.

CTvox 3.3 (Bruker, Billerica, Massachusetts, United States) was used to visualize the 3D structure of the yarn that was reconstructed from 700 slices (cross-section), as shown in Figure 10.

### 3.3. Epoxy Grinding Polishing

**Figure 11:**
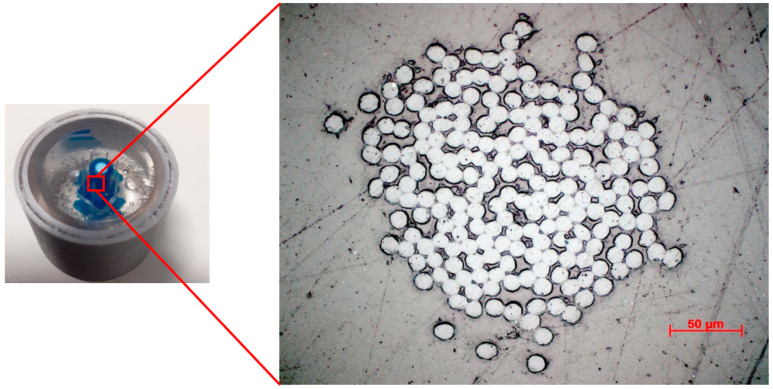
Cross-section image of the polyester yarn obtained by epoxy grinding–polishing method.

On each ground–polished level, eight images of the eight yarns were captured. Figure 11 shows one of these cross-sections. It was noticed that the structure of the individual yarns is clear and of a better visualization compared to the cross-sections obtained via the microtome method. The fibers’ diameter distribution was measured manually by ImageJ, and the mean diameter obtained by µCT was found to be 11.24 ± 0.60 µm. In the literature, a group of researchers reported the use of the epoxy grinding polishing method to image the issue sections instead of using the microtome [28]; however, such a method has not been reported before for the imaging or yarn’s cross-section.

Table 2 shows a brief comparison between the three different methods to obtain yarn’s cross-sectional images.

## 4. Conclusions

The study summarizes a methodological approach using three different methods to obtain the cross-section of a textile yarn so other researchers can select and apply the suitable method for their investigations. The yarn cross-section can reveal important information on the yarn properties, such as yarn’s packing density and yarn’s mechanical strength. The study showed different methods that can be used to obtain the cross-section of a yarn. In conclusion, the microtome and the epoxy grinding–polishing methods can give a clear cross-section; however, a 3D reconstruction of the structure would be very complicated. On the other hand, using a µCT scanner allows the 3D reconstruction, modeling and simulation of the yarn and preserves the complete yarn layers and their sequence; however, a high-resolution scanner is needed to obtain a clear individual fiber’s structure. The epoxy grinding polishing method can be used when clear cross-sectional images are needed as this method gives better contrast than the microtome method; however, the ground layer cannot be imaged once again after being ground. On the other hand, the microtome method can preserve the sliced layers in case the researcher wants to reimage a specific cross-section.

Comparing the three methods, we concluded that the µCT is the fastest method compared to other methods. In addition, it is a nondestructive method that does not destroy the sample’s structure and it allows 3D reconstruction of the scanned sample; however, a high-resolution scanner (the diameter of a single fiber should be represented at least by 20 pixels) is needed to obtain a clear circular cross-section of the fibers. This work can be very useful for researchers as well as industrial partners to know in depth how the cross-section of yarn can be observed using different techniques.

## Figures and Tables

**Table 1:** µCT sample scanning parameters.

Parameter	Value
Source voltage	50 kV
Source current	200 µA
Image pixel size/Scanning resolution	1 µm
Rotation step/angle	0.100°
Size of the projection image	3280 × 1078
Scanning width	22%

**Table 2:** Comparison between the three different methods to obtain a yarn’s cross-section.

Method	Average Fiber Diameter (µm)	3D Reconstruction/Visualization	Average Time to Obtain the Cross-Sections	Type of Test
Microtome	10.96 ± 0.83	Not applicable	5 days (limited number of cross-sections)	Destructive
µCT	10.50 ± 1.13	Applicable	5–6 h for all cross-sections “each of 1 µm thickness” for a 3 mm length sample	Nondestructive
Epoxy grinding–polishing	11.24 ± 0.60	Not applicable	2 days (limited number of cross-sections)	Destructive

## Data Availability

Not applicable.

## References

[1] Abrahart E.N., Whewell C.S. Textile Finishing Processes. https://www.britannica.com/topic/textile/Textile-finishing-processes.

[2] Özkan M., Duru Baykal P., Özkan İ. (2021). Investigation on the Performance Properties of Polytrimethylene Terephthalate (PTT) Based Staple Fibers and Cotton Blended OE-Rotor Yarns. J. Text. Inst..

[3] Wang J., Zhou H., Liu Z., Peng X., Zhou H. (2021). Statistical Modelling of Tensile Properties of Natural Fiber Yarns Considering Probability Distributions of Fiber Crimping and Effective Yarn Elastic Modulus. Compos. Sci. Technol..

[4] Qiu H., Iemoto Y., Tanoue S. (2007). Effects of Cross-Sectional Shape of Yarn Duct of Interlacer on the Properties of Interlaced Yarn. J. Text. Eng..

[5] Long R.L., Delhom C.D., Bange M.P. (2021). Effects of Cotton Genotype, Defoliation Timing and Season on Fiber Cross-Sectional Properties and Yarn Performance. Text. Res. J..

[6] Krucińnska I. (1988). Fiber Blending Irregularities in Cross Sections and on Yarn Surfaces in Relation to Yarn Properties. Text. Res. J..

[7] Zheng S., Zou Z., Shen W., Cheng L. (2012). A Study of the Fiber Distribution in Yarn Cross Section for Vortex-Spun Yarn. Text. Res. J..

[8] Wang Y., Sun X. (2001). Digital-Element Simulation of Textile Processes. Compos. Sci. Technol..

[9] Balokas G., Kriegesmann B., Czichon S., Rolfes R. (2019). Stochastic Modeling Techniques for Textile Yarn Distortion and Waviness with 1D Random Fields. Compos. Part A Appl. Sci. Manuf..

[10] Kaldor J.M., James D.L., Marschner S. Simulating Knitted Cloth at the Yarn Level. Proceedings of the SIGGRAPH’08: International Conference on Computer Graphics and Interactive Techniques, ACM SIGGRAPH 2008 Papers 2008.

[11] McMillan D.B., Harris R.J. (2018). Introduction. An Atlas of Comparative Vertebrate Histology.

[12] Yousif M.Q., Qasem S.A. (2016). Tissue Processing and Staining for Histological Analyses. Skin Tissue Engineering and Regenerative Medicine.

[13] Kilic M., Buyukbayraktar R.B., Kilic G.B., Aydin S., Eski N. (2014). Comparing the Packing Densities of Yarns Spun by Ring, Compact and Vortex Spinning Systems Using Image Analysis Method. Indian J. Fibre Text. Res..

[14] Dey P. (2018). Tissue Microtomy: Principle and Procedure. Basic and Advanced Laboratory Techniques in Histopathology and Cytology.

[15] Yu X.W., Wang H., Wang Z.W. (2018). Analysis of Yarn Fiber Volume Fraction in Textile Composites Using Scanning Electron Microscopy and X-Ray Micro-Computed Tomography. J. Reinf. Plast. Compos..

[16] Lu X., Bertei A., Finegan D.P., Tan C., Daemi S.R., Weaving J.S., O’Regan K.B., Heenan T.M.M., Hinds G., Kendrick E. (2020). 3D Microstructure Design of Lithium-Ion Battery Electrodes Assisted by X-Ray Nano-Computed Tomography and Modelling. Nat. Commun..

[17] Haleem N., Liu X., Hurren C., Gordon S., Najar S.S., Wang X. (2018). Investigating the Cotton Ring Spun Yarn Structure Using Micro Computerized Tomography and Digital Image Processing Techniques. Text. Res. J..

[18] Soltani P., Johari M.S., Zarrebini M. (2015). 3D Fiber Orientation Characterization of Nonwoven Fabrics Using X-Ray Micro-Computed Tomography. World J. Text. Eng. Technol..

[19] Rinaldi R.G., Blacklock M., Bale H., Begley M.R., Cox B.N. (2012). Generating Virtual Textile Composite Specimens Using Statistical Data from Micro-Computed Tomography: 3D Tow Representations. J. Mech. Phys. Solids.

[20] Bale H., Blacklock M., Begley M.R., Marshall D.B., Cox B.N., Ritchie R.O. (2011). Characterizing Three-Dimensional Textile Ceramic Composites Using Synchrotron x-Ray Micro-Computed-Tomography. J. Am. Ceram. Soc..

[21] Depriester D., Rolland du Roscoat S., Orgéas L., Geindreau C., Levrard B., Brémond F. (2022). Individual Fibre Separation in 3D Fibrous Materials Imaged by X-Ray Tomography. J. Microsc..

[22] Toda M., Grabowska K.E. (2013). Computed Microtomography in the Analysis of Fiber Migration in Yarn. Autex Res. J..

[23] Shanbeh M., Hasani H., Manesh F.Y. (2012). An Investigation into the Fatigue Behavior of Core-Spun Yarns under Cyclic Tensile Loading. J. Eng. Fibers Fabr..

[24] Ishtiaque S.M., Mawkhlieng U., Yadav V.K. (2019). Fabric Comfort by Modifying Yarn Structure: Part i—Study on Structural Changes by Cross-Sectional Microtomy of Yarn. Indian J. Fibre Text. Res..

[25] Gharahaghaji A.A., Zargar E.N., Ghane M., Hossaini A. (2009). Cluster-Spun Yarn—A New Concept in Composite Yarn Spinning. Text. Res. J..

[26] Straumit I., Lomov S.V., Wevers M. (2015). Quantification of the Internal Structure and Automatic Generation of Voxel Models of Textile Composites from X-Ray Computed Tomography Data. Compos. Part A Appl. Sci. Manuf..

[27] Naouar N., Vidal-Salle E., Schneider J., Maire E., Boisse P. (2015). 3D Composite Reinforcement Meso F.E. Analyses Based on X-Ray Computed Tomography. Compos. Struct..

[28] Mukhamadiyarov R.A., Sevostyanova V.V., Shishkova D.K., Nokhrin A.V., Sidorova O.D., Kutikhin A.G. (2016). Grinding and Polishing Instead of Sectioning for the Tissue Samples with a Graft: Implications for Light and Electron Microscopy. Micron.

